# Factors associated with the level of occupational stress among nurses—a cross-sectional study

**DOI:** 10.3389/fpubh.2026.1891028

**Published:** 2026-07-22

**Authors:** Katarzyna Lisowicz, Katarzyna Tomaszewska, Dawid Makowicz, Marek Sobolewski, Krystyna Kowalczuk

**Affiliations:** 1 Department of Health, State University of Applied Sciences in Krosno, Krosno, Poland; 2 Faculty of Health Care, Rev. Bronisław Markiewicz State University of Applied Sciences in Jarosław, Jarosław, Poland; 3 Faculty of Management, Rzeszów University of Technology, Rzeszow, Poland; 4 Department of Integrated Medical Care, Medical University of Białystok, Białystok, Poland

**Keywords:** nurses, risk factor, subjective working conditions, workload, work-related stress

## Abstract

**Introduction:**

Occupational stress is a significant issue in the nursing profession, stemming from the high level of responsibility and excessive workload in demanding clinical settings. Prolonged exposure to stress can lead to adverse health effects and a decline in the quality of patient care.

**Objective:**

The aim of the study was to assess the level of occupational stress among nurses and to analyze the relationship between occupational stress and selected sociodemographic and work-related factors.

**Materials and methods:**

The study included 588 male and female nurses employed in three hospitals. A diagnostic survey method was employed, utilizing the Subjective Work Assessment (SWA) Questionnaire and a proprietary questionnaire that covered socio-demographic and work-related data. Statistical analysis was performed using non-parametric tests and linear regression.

**Results:**

In the study group, high levels of work-related stress were found. The highest levels of stress were observed among those working overtime and those with a higher patient-to-nurse ratio. Significant differences were also observed between different places of employment—the highest levels were recorded in intensive care units, while the lowest levels were recorded in primary healthcare. Marital status also influenced stress levels—those in a relationship experienced lower levels of stress. In multivariate analysis, significant predictors of stress included age (positive correlation), length of service (negative correlation), gender, education, work system, and overtime work.

**Conclusions:**

Work-related stress among nurses is a multifactorial phenomenon, with both organizational and sociodemographic factors involved. Higher levels of occupational stress are primarily related to organizational factors, such as workload, patient-to-nurse ratio, and overtime work.

## 1 Introduction

Work-related stress is among the major challenges facing contemporary nursing, impacting both the wellbeing of healthcare staff and the quality and safety of patient care.

Nurses are one of the professions that are particularly prone to long-term stress, due to the nature of their work, which involves constant exposure to illness, suffering, and death. Furthermore, they are responsible for patients' health and lives, often have to make rapid clinical decisions, and work in conditions of staff shortages and limited organizational resources (1, 2). Jennings points out that nursing has been recognized as a highly stressful profession since the 1960s, and the main sources of stress include patient care, decision-making, professional responsibility, and the need to adapt to organizational changes (1).

Stress, as defined by Lazarus, is a particular relationship between a person and their environment, perceived by the person as demanding or exceeding their adaptive resources, and threatening their wellbeing. In the context of nursing, this means that identical working conditions may be interpreted differently by different individuals; however, chronic exposure to occupational stressors significantly increases the risk of burnout, psychosomatic disorders, sick leave and resignation from work (1, 3).

Recent research confirms that the most important determinants of work-related stress among nurses include work overload, staff shortages, shift work, role conflict, limited decision-making autonomy, and insufficient support from superiors (2, 4). In the study by Jaradat and Qtait, all participants reported moderate levels of occupational stress, with the highest scores related to task overload and role conflict. The authors emphasized that this stress originated mainly from systemic and organizational factors, rather than from demographic characteristics of the staff (5). Similar observations were presented by Gilasi et al. (6), who showed that as many as 87.71% of the nurses surveyed experienced high levels of occupational stress, with higher results observed in women.

Stress levels are particularly high in units where staff operate in high-risk clinical environments and under time pressure. A study by Wudarczyk et al. demonstrated a strong correlation between stress levels and coping strategies, as well as with life satisfaction, emphasizing the need to implement systemic psychological and organizational support. A systematic review by Montero-Tejero et al. (7) also found that the main stressors among emergency nurses include work overload, verbal and physical aggression, insufficient resources, and interpersonal stressors, while team support has a protective effect.

Interpersonal relationships and management style are also important. Jennings indicates that conflict with physicians, inappropriate relationships with fellow nurses, lack of support from superiors, and ineffective leadership are significant predictors of psychological stress and burnout. In turn, a study by Dziedzic et al. (8) showed a statistically significant negative correlation between the level of social support and the severity of occupational stress, confirming the protective role of family and professional support. Social support serves as a buffer, reducing the negative effects of stress and improving staff's mental resilience.

Shift work also remains a significant risk factor, particularly the 12-h shift system. Wilczek-Rużyczka et al. (9) demonstrated a significant association between making unintentional errors resulting from 12-h shifts and the severity of occupational burnout in terms of depersonalization and a reduced sense of personal achievement. Prolonged fatigue, sleep disturbances, and chronic psychological stress affect not only nurses' health but also patient safety, increasing the risk of medical errors (1, 9).

The literature also highlights the importance of individual factors, such as age, professional experience, self-esteem and stress management strategies. Older and more experienced nurses are more likely to use active problem-solving strategies, whereas younger staff are more likely to experience heightened stress and adaptive difficulties (10). Moreover, research indicates that higher self-esteem is associated with lower levels of perceived stress and a reduced risk of burnout (11).

Previous research suggests that the severity of work-related stress among nurses can be modified both by socio-demographic and organizational factors. Age, gender, marital status, level of education, and length of service are cited as potential determinants of stress; however, the findings remain inconclusive (2, 5, 6). Some authors point to higher levels of stress among younger nurses and staff with less professional experience, while other studies emphasize the dominant role of work environment factors, such as workload, shift work, staff shortages, and type of ward (1, 4, 8). Particularly high levels of stress are observed in wards where time pressure and clinical responsibility are greatest (4, 6, 10).

Stress in nurses' work is a highly undesirable factor—higher levels of stress naturally have a negative impact on the mental and physical health of the nurses themselves, but the specific nature of their work means that higher levels of stress can lead to a deterioration in relationships with patients, and in extreme cases may even increase the risk of errors in patient care. Moreover, the literature emphasizes that univariate analysis may not reflect the true complexity of occupational stress. Therefore, multivariate models are becoming increasingly important, as they allow for the identification of independent predictors of stress and the assessment of their relative impact (6, 8). This approach allows for more precise planning of organizational and preventive interventions focused on the highest-risk groups. Work-related stress in nursing should therefore be viewed as a multifactorial phenomenon resulting from the interaction of organizational, psychosocial and individual burdens. Its consequences go far beyond the health of on individual person, impacting the functioning of entire therapeutic teams and the quality of healthcare. Identifying factors that exacerbate occupational stress provides the basis for designing effective preventive and interventional measures aimed at improving nurses' working conditions and reducing the risk of burnout (11).

In view of the above, the aim of the study was to assess the level of occupational stress among nurses and to analyze the relationships between occupational stress and selected sociodemographic and work-related factors.

Based on the main objective, specific objectives were formulated:

1. Assessment of occupational stress levels among nurses based on the total stress score and particular dimensions of stress in the SWA questionnaire;
2. Analysis of the relationship between sociodemographic variables (including gender, age, place of residence, marital status, education) and the intensity of occupational stress;
3. Analysis of the co-occurrence of professional factors (length of service, place of employment, type of hospital ward, work system, additional place of work) with the level of occupational stress;
4. Comparison of the level of occupational stress among groups distinguished by the type of ward and work organization;

## 2 Methods

### 2.1 Design of the study

A cross-sectional observational study was conducted using a diagnostic survey method. The study was designed to assess the relationship between selected sociodemographic and occupational factors and the level of work-related stress among nurses. The cross-sectional design enabled the simultaneous analysis of socio-demographic, organizational, and occupational variables, as well as the assessment of the total measure of stress and its individual dimensions in a large, diverse group of nurses. The study was quantitative in nature and was based on the analysis of data obtained using a standardized psychometric tool—the SWA questionnaire—and a proprietary survey questionnaire covering sociodemographic data and information on working conditions. The study was designed in accordance with the Strengthening the Reporting of Observational Studies in Epidemiology (STROBE) guidelines (12).

### 2.2 Place and time of the study

The study was conducted in three hospitals located in the Podkarpackie Voivodeship from December 2025 to the end of February 2026. The facilities were selected intentionally, based on their management's consent to participate in the project. In each hospital, surveys were distributed to departments of various specialties in order to include diverse work environments and increase the heterogeneity of the study sample.

### 2.3 Study participants

A purposive sampling strategy was used to select the participating healthcare institutions. Within the hospital wards included in the study, all nurses who met the inclusion criteria and were available during the data collection period were invited to participate. Approximately 200 completed questionnaires were planned to be obtained from each hospital, corresponding to the target sample size of 600 participants. Ultimately, 588 correctly completed questionnaires were included in the analysis, representing 98.0% of the planned sample size. The numbers of respondents recruited from individual institutions were as follows: Hospital A−200 participants, Hospital B−182 participants, and Hospital C−196 participants.

Participation in the study was voluntary and anonymous. Eligible participants were registered nurses actively engaged in professional practice during the study period and who provided informed consent to participate. Nurses working under different work schedule systems were included, including single-shift, two-shift, and three-shift schedules involving night duties. In Poland, extended working-time arrangements, including 12-h shifts, are permitted in healthcare institutions in accordance with applicable labor laws and healthcare regulations.

Individuals who declined participation or failed to complete the questionnaire sufficiently to allow data analysis were excluded from the study. Due to the anonymous nature of the survey, no records were maintained regarding the total number of nurses invited to participate or those who refused participation. Consequently, it was not possible to calculate the response rate. The questionnaires were sent to cooperating hospitals with a request to be completed.

### 2.4 Research tools

The study used a proprietary questionnaire covering sociodemographic, occupational, and organizational variables, such as age, gender, place of residence, marital status, education level, total length of service, length of service at the current workplace, place of employment, type of ward, work schedule, number of working hours per week, additional place of employment, and patient-to-nurse ratio on duty. These variables were considered potential factors influencing occupational stress levels and were included in the statistical analysis.

To assess the level of work-related stress, the standardized Subjective Work Assessment Questionnaire (SWAQ) by Dudek, Waszkowska and Hanke, developed at the Institute of Occupational Medicine named after Prof. J. Nofer in Łódz, was used (13). The tool investigates the respondents' subjective perception of work and measures their individual feeling of occupational stress and exposure to psychosocial hazards in the work environment. The questionnaire consists of 55 questions (in some studies described as 55–57 items) covering various aspects of work that are rated by the respondents on a 5-point scale to indicate the extent to which a given situation is burdensome, irritating, or stressful. “1” means that the respective stressor is absent or negligible and “5” indicates high intensity of the stressor and experiencing severe stress. The total score is an indicator of the overall occupational stress—the higher the score, the greater the severity of the stress. The questionnaire also allows for the analysis of specific dimensions of work-related stress, such as mental workload, lack of rewards, organizational uncertainty, social interaction, sense of threat, physical strain, unpleasant working conditions, and lack of control. This enables both an overall assessment of stress and recognition of the most stressful areas of the work environment.

The tool demonstrates good psychometric validity. The internal consistency of the questionnaire, as measured by Cronbach's alpha, is considered satisfactory and good for population-based studies (α > 0.70). This confirms that the questionnaire can be useful in the assessment of occupational stress in health professionals. The SWAQ is widely used in survey studies of nurses and other occupational groups exposed to chronic work-related stress.

The SWAQ questionnaire was selected due to its wide application in Polish studies concerning psychosocial working conditions and its ability to comprehensively assess various dimensions of occupational stress. The instrument was developed and validated for Polish conditions, which increases its usefulness in studies involving nursing personnel. Permission to use the questionnaire was obtained. The assessment of occupational stress was based solely on a self-report method. No objective physiological indicators of stress or methods for assessing physical workload (e.g., the Åstrand or Ramanathan classifications) were used in the study. The aim of the study was to analyze subjectively perceived occupational stress and psychosocial working conditions.

### 2.5 Data collection procedure

After obtaining permission from the management of the institutions, paper questionnaires were provided to the study coordinators in the participating hospitals. They were then distributed directly to nurses who met the inclusion criteria. Completed questionnaires were returned in sealed envelopes, which ensured the anonymity of the respondents. The questionnaires, in paper form, were distributed to nurses employed in healthcare facilities included in the study. Participation in the study was entirely voluntary and anonymous. Each respondent was informed about the purpose of the study and its scientific nature, as well as about principles of confidentiality and anonymity of the collected data. Moreover, the respondents could withdraw from the study at any time without giving any reason.

### 2.6 Statistical analysis

Statistical analysis was conducted with IBM SPSS Statistics version 29.0. The level of statistical significance was set at *p* < 0.05. In the first stage of the analysis, descriptive statistics were calculated for the studied variables, including the arithmetic mean (M), median (Me), standard deviation (SD), minimum and maximum values, and quartiles. To assess differences in occupational stress between 2 study groups (categorized according to gender, additional workplace, place of residence), the Mann–Whitney test, a nonparametric counterpart to the *t*-test for independent samples, was used. To compare more than two study groups (defined by marital status, education level, place of employment, work system), the Kruskal–Wallis test was used. Non-parametric tests were employed because the assumptions of normality of distribution and homogeneity of variance were not met. Spearman's rank correlation coefficient (rS) was used to assess the relationship between the level of work-related stress and numerical variables, such as age, total length of service and length of service at the current place of employment. Age was analyzed both as a continuous variable and within several age groups (< 30, 31–40, 41–50, 51–60, and >60 years). A division into individuals below and above 40 years of age was not applied because the classification used allowed for a more detailed assessment of age-related associations and stages of professional career development. This coefficient allows for the assessment of the strength and direction of the monotonic relationship between the analyzed variables and is robust to the presence of outliers. In order to identify independent predictors of the overall measure of work-related stress, a multivariate analysis was conducted using a linear regression model (13–15). Prior to performing the linear regression analysis, the fulfillment of the model's basic assumptions was assessed, including the linearity of the relationship, the normality of the residual distribution, and the absence of significant multicollinearity between the independent variables. No significant violations of the regression assumptions were found. The sample size was deemed sufficient to conduct multivariate analyses in accordance with the methodological recommendations for linear regression. Only complete questionnaires were included in the analysis; therefore, the analysis did not account for missing data. The model included sociodemographic and occupational variables that were statistically significant in univariate analyses or were of significant clinical and organizational importance.

### 2.7 Ethical approval

The study was conducted in accordance with the principles of the Declaration of Helsinki. Approval was obtained from the PANS Bioethics Committee in Krosno: Resolution No. 16/2025.

## 3 Results

### 3.1 Study sample characteristics

The study included 588 nurses. The vast majority of the study group were women (83.2%), while men accounted for 16.8% of the respondents. The largest age group were those aged 51–60 years (29.9%), followed by those aged 31–40 (25.0%) and 41–50 (23.0%). Respondents under 30 constituted 18.2% of the study sample, while those over 60 were the smallest group (3.9%).

Slightly more than half of the respondents (50.7%) lived in rural areas and 36.6% lived in towns with up to 50,000 inhabitants. Respondents living in larger towns and cities constituted a significantly lower percentage of the study population. Most respondents reported being married (65.6%), 15.5% were single. Divorced persons accounted for 7.8%, widows and widowers for 4.6%, and those in a relationship for 6.5%. As regards education level, the majority held a master's degree (48%) or a bachelor's degree (36%). 14% of the respondents had graduated from a secondary nursing school, while those with postgraduate studies and doctorates constituted a small percentage of the study group. The most common places of employment were medical (37.1%) or surgical (31.0%) hospital wards. 16.0% of the respondents worked in intensive care units, and another 16.0% worked in primary healthcare facilities.

Most of the survey participants were employed on full-time contracts (88.8%). The most commonly reported work schedule was a two-shift system (60.0%), followed by a single-shift system (22.8%) and a three-shift system with night shifts (17.0%). Over half of the respondents worked between 40 and 59 h per week (57.1%), and 34.0% of those surveyed reported working in more than one workplace. The number of working hours per week referred to the total working time regardless of the number of days worked per week. Due to different forms of employment and work organization systems, the number of working days could vary among respondents.

The average number of patients per nurse on duty was 15.1, with the median number of 13. 52.4% of the respondents occasionally worked overtime, and 40.8% reported no overtime work. 38.9% of the respondents took a sick leave during the last year, with its duration ranging most commonly from 1 to 7 days. Particular results are presented in Table 1.

**Table 1:** Sociodemographic and occupational characteristics of the study sample (*N* = 588).

Characteristic	Category	*n*	%
Gender	Female	489	83.2
	Male	99	16.8
Age	< 30 years	107	18.2
	31–40 years	147	25.0
	41–50 years	135	23.0
	51–60 years	176	29.9
	>60 years	23	3.9
Place of residence	Rural area	298	50.7
	Town up to 50 thousand inhabitants	215	36.6
	Town 50–200 thousand inhabitants	68	11.6
	City >200 thousand inhabitant	7	1.2
Marital status	Single	91	15.5
	In a relationship	38	6.5
	Married	386	65.6
	Divorced	46	7.8
	Widowed	27	4.6
Education level	Secondary nursing school	85	14.0
	Bachelor's degree	211	36.0
	Master's degree	283	48.0
	Postgraduate diploma	24	4.0
	Doctorate	3	1.0
Place of employment	Medical hospital ward	218	37.1
	Surgical hospital ward	182	31.0
	Intensive care unit	94	16.0
	Primary healthcare	94	16.0
Work system	Single-shift	134	22.8
	Two-shift	353	60.0
	Three-shift	100	17.0
Additional work place	Yes	200	34.0
	No	383	65.1

Source: own work.

### 3.2 Results of the subjective work assessment questionnaire (SWAQ)

The analysis of the relationship between gender and work-related stress revealed statistically significant differences between women and men. In all analyzed dimensions of the SWAQ, as well as in the overall stress measure, men scored higher than women, which indicates a higher level of occupational stress in this group. The largest differences were observed for overall stress, mental workload, lack of rewards at work, social interaction and lack of support. However, it should be noted, that the group sizes were clearly unequal—women made up the vast majority of the study population (*N* = 489), while the number of men was significantly smaller (*N* = 99). Therefore, the observed differences must be interpreted with caution, because such an inequity in group sizes may affect comparability of the results and the power of statistical inference. Nevertheless, the findings of the study suggest that gender may be an important factor determining the level of occupational stress in nursing staff. It should also be emphasized that the gender structure of the study group reflects the actual occupational structure of the nursing profession in Poland, where women constitute the vast majority of nursing personnel.

For age and length of service, the analysis was carried out using Spearman's rank correlation coefficient. No significant correlations were found between age and most of the analyzed dimensions of work-related stress. Only for “unpleasant working conditions” a statistically significant, albeit very weak, negative correlation was observed, with no practical meaning. Similar results were obtained for “length of service,” which—both in total and for the current employer—showed no correlation with the level of work-related stress. Although a few statistically significant correlations were observed for length of service in total, their strength was very low, which means this factor cannot be considered a significant predictor of stress. Ultimately, it can be concluded that neither age nor length of service significantly influenced the level of work-related stress among the surveyed nurses.

The analysis of “place of residence,” comparing those living in rural areas vs. those living in urban areas, revealed no statistically significant differences in any of the dimensions of occupational stress or in the overall stress measure. Both the level of mental strain and other components of occupational stress were comparable between these study groups. No relationship was found between place of residence and the severity of occupational stress.

The analysis of the relationship between work-related stress and marital status was carried out in three groups: singles (single men and women), those in a relationship (whether cohabiting or married), and those whose relationship had ended as a result of divorce or widowhood. Combining individual categories into bigger marital status groups improved the reliability of the comparisons.

The results indicated a very clear, statistically significant differences in stress levels between the analyzed groups. The differences were seen in all dimensions rated in the SWA questionnaire. The most favorable results were recorded among those in a relationship—for example, the median total stress level in this group was 134 points vs. 147 points in singles and as many as 168 points in singles whose relationship had ended. These findings suggest that personal and relationship circumstances may be associated with the nurses' mental wellbeing in the workplace. Those in stable relationships demonstrate greater resilience to stress, which is also an important message from the perspective of employers and work organization (Table 2).

**Table 2:** Marital status and the level of work-related stress.

Subjective work assessment	Marital status									*p*
	Singles (*N* = 91)			Persons in a relationships (*N* = 424)			Relationship breakdown (*N* = 73)			
	Mean	Me	Std. dev.	Mean	Me	Std. dev.	Mean	Me	Std. dev.	
Overall stress	154.9	147	46.4	140.8	134	48.0	168.0	165	51.4	0.0000^***^
Mental workload	25.3	25	8.1	22.5	21	8.7	27.7	29	9.3	0.0000^***^
Lack of rewards	22.3	22	7.9	20.3	19	7.7	25.2	25	7.6	0.0000^***^
Organizational uncertainty	19.7	18	6.6	19.1	18	6.9	21.9	21	7.0	0.0048^**^
Social interaction	13.8	12	5.2	12.8	11	4.6	15.1	14	5.2	0.0013^**^
Sense of threat	14.5	14	4.5	13.4	13	4.7	15.7	16	5.3	0.0008^***^
Physical strain	11.5	12	5.1	9.9	8	5.4	11.8	12	5.9	0.0022^**^
Unpleasant working conditions	8.5	9	3.5	7.6	6	3.7	8.9	9	3.9	0.0032^**^
Lack of control	11.1	10	3.9	10.2	9	3.8	11.7	10	4.0	0.0022^**^
Lack of support	7.8	8	3.4	6.8	6	3.1	8.4	9	3.2	0.0000^***^
Sense of responsibility	11.8	11	3.7	10.7	10	3.9	12.2	12	4.1	0.0014^**^

Source: own work. *p*, test probability values calculated with the Kruskal–Wallis test. The symbols next to the p-values indicate the level of statistical significance.

The analysis of stress intensity differences by education level was conducted by dividing the participants into three groups: those who graduated from secondary nursing school, those with a bachelor's degree, and those with a master's degree. The group of participants with a master's degree included also a relatively small number of participants with postgraduate education and an even smaller number of nurses with a PhD. The reason for such a categorization was the need to increase the analyzed group sizes and improve the validity of the comparative analyses.

The results indicate that educational attainment has a major impact on the severity of overall stress and almost all of its dimensions, with the exception of the sense of lack of control and physical strain, where no significant differences were observed. The highest level of stress was found among those with a bachelor's degree, and the lowest one in the group with secondary education. Median total stress score in the bachelor's degree group was 151 vs. 137 in the master's degree group and 117 in the secondary education group. The results suggest that there is a relationship between education level and coping with stress (Table 3).

**Table 3:** Education and the level of work-related stress.

Subjective work assessment	Education									*p*
	Secondary nursing school (*N* = 85)			Bachelor's degree (*N* = 211)			Master's degree (*N* = 291)			
	Mean	Me	Std. dev.	Mean	Me	Std. dev.	Mean	Me	Std. dev.	
Overall stress	137.9	117	56.4	155.1	151	50.4	142.5	137	44.9	0.0028^**^
Mental workload	21.7	19	10.4	25.2	25	8.7	23.0	22	8.3	0.0009^***^
Lack of rewards	19.3	16	9.1	22.2	22	8.0	21.0	20	7.3	0.0073^**^
Organizational uncertainty	18.9	17	7.2	20.5	20	7.1	18.9	18	6.7	0.0241^*^
Social interaction	12.9	11	5.3	14.1	13	5.3	12.7	12	4.3	0.0114^*^
Sense of threat	13.2	11	5.5	14.6	15	4.9	13.4	13	4.5	0.0071^**^
Physical strain	9.5	8	6.1	10.8	12	5.5	10.3	12	5.2	0.0786
Unpleasant working conditions	7.0	6	3.7	8.5	9	3.8	7.7	6	3.7	0.0029^**^
Lack of control	10.5	9	4.2	11.1	10	4.2	10.2	9	3.5	0.1736
Lack of support	6.8	6	3.3	7.8	8	3.4	6.8	6	2.9	0.0051^**^
Sense of responsibility	10.5	9	4.5	11.6	11	3.9	10.7	10	3.6	0.0122^*^

Source: own work. *p*, test probability values calculated with the Kruskal–Wallis test. The symbols next to the p-values indicate the level of statistical significance.

The overall stress level and particular dimensions of stress show significant differences depending on the profile of hospital wards. Based on the total stress measure, it can be concluded that the level of work-related stress among nurses from intensive care units (median score of 163.5) unfavorably outdistances the level of stress in other work places. The stress levels are much lower, and similar to each other, in the remaining types of hospital wards (139.5 points in medical wards, 137 points in surgical wards), while working in primary healthcare is associated with the lowest stress burden (median score of 122.5)—Table 4.

**Table 4:** Place of employment and the level of work-related stress.

Subjective work assessment	Place of employment								*p*
	Medical hospital ward (*N* = 218)		Surgical hospital ward (*N* = 182)		Intensive care unit (*N* = 94)		Primary healthcare (*N* = 94)		
	Mean	Me	Mean	Me	Mean	Me	Mean	Me	
Overall stress	147.4	139.5	142.3	137	159.2	163.5	139.0	122.5	0.0107^*^
Mental workload	23.6	22	23.2	22	25.2	27.5	23.0	21	0.2156
Lack of rewards	21.2	20	20.7	20	22.8	24	20.6	18	0.1288
Organizational uncertainty	19.8	19	19.1	18	21.0	21	18.0	16	0.0181^*^
Social interaction	13.3	12	12.7	11	14.5	14	12.9	11	0.0297^*^
Sense of threat	14.1	13	13.5	13	15.2	15	12.6	12	0.0006^***^
Physical strain	10.6	12	9.9	8	11.6	12	9.7	8	0.0368^*^
Unpleasant working conditions	8.1	9	7.5	6	9.0	9	7.1	6	0.0011^**^
Lack of control	10.5	9	10.0	8	11.6	11	10.4	9	0.0033^**^
Lack of support	6.9	6	6.9	6.5	8.0	8	7.4	7	0.0188^*^
Sense of responsibility	11.3	11	10.8	10	11.7	12	10.1	9	0.0176^*^

Source: own work. *p*, test probability values calculated with the Kruskal–Wallis test. The symbols next to the p-values indicate the level of statistical significance.

The analysis of the relationship between work patterns (single-, two-, and three-shift system) and the intensity of stress and its individual dimensions among the surveyed nurses indicates that work system does not significantly differentiate stress levels, especially when compared to other factors, such as ward type or sociodemographic characteristics. In the case of total stress, differences were at the borderline of statistical significance (*p* = 0.0501). The highest stress levels were observed in those working in a two-shift system, while the lowest in a three-shift system, which may be surprising. Nurses working in a single-shift system achieved intermediate results.

The differences were more pronounced for selected dimensions of stress. Statistically significant differences were observed for organizational uncertainty, a sense of threat, unpleasant working conditions, and a sense of responsibility. In all these areas, the highest scores were reported in the group working in a two-shift system. A particularly strong correlation was observed for unpleasant working conditions (*p* = 0.0001), indicating that this aspect of the work environment may be particularly burdensome in this system. The statistically significant differences were seen in organizational uncertainty (*p* = 0.0256), a sense of threat (*p* = 0.0136), and a sense of responsibility (*p* = 0.0402), with the highest stress levels in the two-shift system. The work system shows limited but noticeable correlations with some aspects of stress, especially with factors related to work organization and work environment. The most unfavorable stress profile is observed in two-shift work.

The analysis of overtime work shows a significantly higher level of stress in the overtime group, both in terms of overall stress and in terms of particular dimensions of the nurses' professional activity. The median total stress score in this group is 144 points, compared to 130.5 points in the group without overtime work, and this difference is statistically significant (*p* = 0.0002). Weaker but still statistically significant differences were seen for physical strain (*p* = 0.0398) and unpleasant working conditions (*p* = 0.0064). The results clearly show that nurses working overtime experience higher levels of overall stress than others. The median total stress score in this group is 144.0 points vs. 130.5 points in the group without overtime work, with the difference being statistically significant (*p* = 0.0002; Table 5).

**Table 5:** Overtime work and the level of work-related stress.

Subjective work assessment	Overtime work						*p*
	No (*N* = 240)			Yes (*N* = 345)			
	Mean	Me	Std. dev.	Mean	Me	Std. dev.	
Overall stress	137.9	130.5	50.1	152.2	144	47.5	0.0002^***^
Mental workload	22.1	21	8.9	24.7	24	8.7	0.0004^***^
Lack of rewards	20.0	19	8.1	22.0	22	7.7	0.0013^**^
Organizational uncertainty	18.4	17	7.0	20.3	19	6.8	0.0006^***^
Social interaction	12.5	11	4.8	13.8	12	4.8	0.0007^***^
Sense of threat	13.0	12	4.7	14.4	14	4.8	0.0002^***^
Physical strain	9.9	8	5.6	10.7	12	5.4	0.0398^*^
Unpleasant working conditions	7.4	6	3.7	8.2	9	3.8	0.0064^**^
Lack of control	10.0	8	4.0	10.9	10	3.7	0.0002^***^
Lack of support	6.6	6	3.2	7.5	7	3.1	0.0001^***^
Sense of responsibility	10.5	10	3.9	11.4	11	3.9	0.0046^**^

Source: own work. *p*, test probability values calculated with the Kruskal–Wallis test. The symbols next to the p-values indicate the level of statistical significance.

The analysis of the relationship between the average number of patients per nurse on duty and the level of work-related stress (overall and according to its particular dimensions) revealed that the level of perceived stress increased along with the growing number of patients. A statistically significant, albeit moderate, relationship was observed for total stress (*r* = 0.14; *p* = 0.0007), indicating that a higher patient load was associated with higher overall stress.

The strongest relationship was seen for mental workload (*r* = 0.17; *p* < 0.001), suggesting that the number of patients under care has a particular impact on psychological wellbeing at work (Table 6).

**Table 6:** Workload and the level of work-related stress.

Subjective work assessment	Average number of patients per nurse on duty
Overall stress	0.14 (*p* = 0.0007^***^)
Mental workload	0.17 (*p* = 0.0000^***^)
Lack of rewards	0.15 (*p* = 0.0004^***^)
Organizational uncertainty	0.08 (*p* = 0.0461^*^)
Social interaction	0.11 (*p* = 0.0068^**^)
Sense of threat	0.09 (*p* = 0.0269^*^)
Physical strain	0.13 (*p* = 0.0018^**^)
Unpleasant working conditions	0.10 (*p* = 0.0228^*^)
Lack of control	0.14 (*p* = 0.0011^**^)
Lack of support	0.15 (*p* = 0.0002^***^)
Sense of responsibility	1.07 (*p* = 0.0808)

Source: own work. The symbols next to the p-values ​​indicate the level of statistical significance.

Results of the linear regression analysis of overall stress with relation to selected sociodemographic and work-related variables has shown that several variables are significantly associated with the stress levels: age is positively related with stress (*B* = 1.3; *p* = 0.0118; ß = 0.30), which means that the severity of stress increases with age. Length of service in total shows the opposite relationship (*B* = −1.2; *p* = 0.0107; ß = −0.31), which means that greater professional experience is associated with lower stress levels. As regards gender: men report higher stress levels than women (*B* = 13.7; *p* = 0.0107), although the magnitude of this effect is relatively small (ß = 0.10). Marital status: individuals in a relationship report significantly lower stress levels than single persons (*B* = −17.2; *p* = 0.0001; ß = −0.16). Education: having a bachelor's degree is associated with higher stress levels compared to the other groups (*B* = 8.9; *p* = 0.0348). Work system: Two-shift work is associated with higher stress (*B* = 11.9; *p* = 0.0035; ß = 0.12). Overtime work also increases stress levels (*B* = 12.9; *p* = 0.0014; ß = 0.13). In terms of the effect size (ß values), age and length of service are the most important (although they work in opposite directions), and are followed by marital status and overtime work (Table 7).

**Table 7:** Multivariate analysis.

Independent factors	Overall stress *R*^2^ = 9.9% *F* = 8.9 *p* = 0.0000^***^		
	*B* (95% CI)	*p*	*ß*
Age [years]	1.3 (0.3; 2.3)	0.0118^*^	0.30
Length of service in total [years]	−1.2 (−2.2; −0.3)	0.0107^*^	−0.31
Gender (males vs. females)	13.7 (3.2; 24.2)	0.0107^*^	0.10
Marital status (in a relationship vs. singles)	−17.2 (−26.1; −8.4)	0.0001^***^	−0.16
Education level (bachelor's degree vs. other)	8.9 (0.6; 17.2)	0.0348^*^	0.09
Work system (two-shift vs. other)	11.9 (3.9; 19.8)	0.0035^**^	0.12
Overtime work (yes vs. no)	12.9 (5.0; 20.8)	0.0014^**^	0.13

Source: own work. *R*^2^, coefficient of determination; *F*, test statistic and *p* value for significance of whole model; *B*, regression coefficient with 95% confidence interval; *p* value for significance of each regression coefficient; ß, standardize regression coefficient. The symbols next to the p-values ​​indicate the level of statistical significance.

The analysis of the SWAQ results showed that in 466 nurses (79.3%), the level of occupational stress exceeded the normative values established for the instrument used, whereas in 122 individuals (20.7%), the score was within the normal range. The stress level in the surveyed nurses is significantly related to individual factors (age, gender) and occupational factors (length of service, work system, overtime), with greater professional experience and being in a relationship having a protective effect, while overtime work and less comfortable working conditions increasing the stress level.

## 4 Discussion

One of the most important findings of the present study was that as many as 79.3% of nurses obtained scores exceeding the normative values of the SWAQ questionnaire, indicating a very high prevalence of occupational stress in the study group. The obtained results confirm that occupational stress among nurses is multifactorial in nature and remains strongly associated with both work organization and selected sociodemographic characteristics. Such a high proportion of individuals experiencing occupational stress may have significant consequences both for the health of nursing personnel and for the quality and safety of patient care. Our findings are consistent with the study by Wudarczyk et al. (6), in which more than 40.0% of nurses experienced a high level of stress. Similar observations were reported by Jaradat and Qtait, who indicated that occupational stress is mainly organizational in origin, with workload and role conflict being the key stressors (2). Studies by Reshia et al. also indicate that moderate and high levels of stress affect the majority of nurses working in high-intensity care environments (16–19). In the present study, the highest stress levels were observed among intensive care unit personnel, which may result from the specific nature of work in these units. ICU nurses provide care to patients in life-threatening conditions who require continuous monitoring and rapid responses to changing clinical conditions. An additional source of burden is frequent exposure to patient death, responsibility for operating advanced medical equipment, and the need to make decisions in situations involving high clinical risk. The obtained results therefore confirm that a high level of stress is a common phenomenon independent of geographical context, indicating its systemic nature.

One of the most important findings of the study was the demonstration that overtime work and an excessive number of patients assigned to one nurse significantly increase the level of stress. These results are consistent with the systematic review by Montero-Tejero et al. (4), which identified workload and staffing shortages as the main determinants of occupational stress. The observed relationship between overtime work and a higher level of occupational stress may be explained by the accumulation of physical and psychological burdens and the reduction of time available for recovery. Long-term work beyond standard working hours promotes chronic fatigue, sleep disturbances, and deterioration of work–life balance. Consequently, it may lead to increased emotional tension, reduced job satisfaction, and greater susceptibility to burnout.

Other studies also point to the dominant role of workload. Gilasi et al. (5) demonstrated that more than 80% of nurses experience high levels of stress, mainly due to excessive job demands. Similar conclusions were presented by Subih et al. (9), who demonstrated a positive relationship between stress levels and a high patient-to-nurse ratio. An increase in the number of patients assigned to one nurse is associated with a greater workload, the need to perform numerous tasks within a limited time, and more frequent decision-making under time pressure. Such work organization may lead to a feeling of lack of control over performed tasks and increase concerns regarding the quality and safety of provided care. Work schedule organization was also of significant importance. In the regression model, overtime work was an independent predictor of stress, which is consistent with the findings of Wilczek–Rużyczka et al. (8), indicating the negative impact of shift work on stress and burnout levels. Studies by Grochowska et al. (20) also emphasize that shift work and excessive duties lead to psychological and physical exhaustion.

In univariate analyses, lack of social support was a significant factor associated with stress levels. These findings are consistent with the study by Dziedzic et al. (7), which demonstrated a significant negative relationship between social support and stress (*r* = −0.21). The importance of interpersonal relationships is also emphasized by Tjugum et al. (10), who indicated that psychosocial factors such as interdisciplinary cooperation and the quality of team relationships have a significant impact on occupational stress levels. Team support therefore serves a buffering function, reducing the negative consequences of occupational burdens.

In the present study, a significant influence of some sociodemographic variables, particularly marital status, was demonstrated. However, it should be emphasized that family burden, number of children, and caregiving responsibilities toward older adults were not directly assessed in this study. Therefore, it is not possible to clearly determine the impact of these factors on perceived stress levels. Individuals who were in a relationship were characterized by lower levels of stress, confirming the protective role of emotional support. Similar findings were reported by Pereira dos Santos et al. (21), who indicated higher stress levels among single individuals. The lower stress levels observed among individuals in a relationship may be related to the protective role of social support. The presence of a life partner facilitates more effective coping with occupational burdens through the possibility of receiving emotional and instrumental support as well as a greater sense of security, which may increase psychological resilience and reduce the negative effects of chronic occupational stress. This mechanism is consistent with the buffering theory of social support in relation to stress. At the same time, the literature remains inconclusive, as some studies have not demonstrated significant relationships between stress and demographic variables (2, 22, 23), suggesting that their impact may depend on the organizational context. It is worth emphasizing that in the present study, men reported higher levels of stress, which differs from the findings of Gilasi et al. (5), where higher values were observed among women. This may result from cultural differences, sample structure, or differences in the perception of stress.

In the univariate analysis, neither age nor length of service showed significant relationships with stress levels; however, in the multivariate model, age proved to be a positive predictor of stress, whereas length of service was a protective factor. This may indicate the coexistence of these variables and a mutual masking effect of their influence in univariate analyses. These findings partially confirm the observations of Wudarczyk et al. (6), indicating the importance of experience and stress coping strategies. Similar relationships were described by Subih et al. (9), who demonstrated that older and more experienced nurses more frequently use adaptive stress-coping strategies. At the same time, other studies have not confirmed significant relationships in this area. The regression analysis results indicate that organizational factors, particularly overtime work and the organization of working time, are significantly associated with occupational stress levels. At the same time, the relatively low coefficient of determination (*R*^2^ = 9.9%) indicates that the analyzed model explains only a small proportion of the variability in occupational stress levels. This suggests that modifying working conditions may represent a more effective direction for preventive actions than interventions focused solely on individual staff characteristics (1, 2). Similar conclusions have been presented by authors studying burnout, emphasizing that stress and burnout result from the interaction of numerous environmental and personal variables (17, 24, 25).

The obtained results suggest that organizational factors, particularly workload and work schedule organization, may play a greater role in shaping occupational stress levels than most of the analyzed sociodemographic variables. These findings are consistent with the current state of knowledge and highlight the need to implement comprehensive preventive measures.

## 5 Conclusions

1. Higher levels of occupational stress were observed among nurses working overtime and caring for a greater number of patients.
2. The highest stress levels were recorded among intensive care unit personnel, whereas the lowest levels were observed in primary healthcare settings.
3. Being in a relationship and having a longer length of service were associated with lower levels of occupational stress.
4. Organizational factors demonstrated a stronger association with occupational stress levels than most of the analyzed sociodemographic variables.

## 6 Practical implications

The results indicate the need to take actions aimed primarily at improving the organization of nurses' work. It is crucial to reduce work overload by aligning staffing to actual needs and by planning shift rosters effectively. Another important element is the reduction of overtime, which is one of the main factors contributing to increased stress levels. Another key area for intervention is strengthening social support in the workplace. Preventive measures should also include individual support for nurses, such as programmes designed to develop stress management skills, mental resilience, and a healthy work-life balance. Implementing comprehensive solutions in this area can help improve staff wellbeing, reduce the risk of burnout, and increase the quality and safety of patient care. In practice, this means the need to monitor the patient-to-nurse ratio, limit excessive overtime work, implement psychological support programs for staff, and develop mentoring initiatives for younger employees. Regular training in stress management as well as strengthening teamwork and support from supervisors may also be beneficial.

## 7 Limitations of the study

The main limitations of the study include its cross-sectional nature, which does not permit drawing conclusions on the cause-and-effect relationships. The use of self-report methods carries the risk of subjective assessment and desirability bias. Unequal sizes of some groups, particularly in terms of gender, may limit the comparability of results. Furthermore, the study was conducted in selected facilities within a single geographical region, which may affect the generalizability of the results. The low proportion of variance explained in the regression model suggests that there are other, unaccounted-for factors that influence the level of work-related stress. In future studies, psychosocial variables, such as coping strategies and personality traits, are worth considering. Another limitation is the fact that differences between individual hospitals were not analyzed because the data were collected in a manner that prevented the identification of specific institutions. However, it should be emphasized that organizational factors and the characteristics of the work environment may influence the level of perceived occupational stress; therefore, this issue requires further investigation. Furthermore, a limitation of the study is the lack of objective measurements of physical and psychophysiological workload. The assessment was based solely on the subjective evaluation of occupational stress and working conditions. In future studies, it would be justified to combine psychometric methods with objective indicators of workload, such as heart rate monitoring, assessment of energy expenditure, or workload severity classifications, which would allow for a more comprehensive evaluation of the relationship between occupational stress and work demands. An additional limitation of the study was the failure to consider certain potentially important variables, such as the number of children, caregiving responsibilities toward older adults, family situation, commuting time to work, or other non-occupational sources of psychological burden. Objective indicators of stress and physiological workload were also not analyzed. Future studies should consider both psychosocial and physiological determinants of occupational stress, which would allow for a more comprehensive assessment of the phenomenon under investigation.

## Data Availability

The original contributions presented in the study are included in the article/supplementary material, further inquiries can be directed to the corresponding author.
